# Assessment of Intravoxel Incoherent Motion Diffusion‐Weighted Imaging in Insomnia

**DOI:** 10.1002/brb3.70785

**Published:** 2025-08-27

**Authors:** Wenjie Tang, Asta Debora, Shouliang Miao, Boli Lin, Qun Huang, Zhengzhong Yuan, Yunjun Yang, Nengzhi Xia

**Affiliations:** ^1^ Department of Radiology The First Affiliated Hospital of Wenzhou Medical University Wenzhou China; ^2^ Department of Traditional Chinese Medicine The First Affiliated Hospital of Wenzhou Medical University Wenzhou China; ^3^ Department of Nuclear Medicine The First Affiliated Hospital of Wenzhou Medical University Wenzhou China

**Keywords:** Insomnia disorder, Magnetic resonance imaging, Intravoxel incoherent motion, Perivascular spaces, Support vector machine

## Abstract

**Purpose:**

Few studies have investigated insomnia using intravoxel incoherent motion imaging. The aim ofthis study was to evaluate the classification performance of intravoxel incoherent motion diffusion‐weighted imaging in differentiating between insomnia disorder patients and normal control subjects. The potential effects of microstructure and microcirculation between sleep quality and glymphatic system function were subsequently explored.

**Method:**

Forty‐two patients with insomnia disorder and 42 normal controls were included in this study. The demographic characteristics, Pittsburgh Sleep Quality Index scores, and imaging data of the subjects were collected. Magnetic resonance imaging revealed enlarged perivascular spaces that indirectly reflect the glymphatic system. Imaging data were subjected to postprocessing, and parameters including the pure diffusion coefficient, pseudo diffusion coefficient and perfusion fraction were calculated. Partial correlation analysis and mediation analysis were used to explore the role of microcirculation between sleep quality and the perivascular space.

**Findings:**

There were brain region‐specific differences between two groups, mainly involving perfusion parameters. D* and *f* in the dorsal–lateral superior frontal gyrus, hippocampus, caudate nucleus, lenticular nucleus, thalamus, amygdala, and fusiform gyrus demonstrated significant differences compared to the healthy group. The support vector machine classifier based on intravoxel incoherent motion imaging parameters has good diagnostic efficiency, with an area under the curve of 0.92. In the mediation analysis, D* in the left thalamus mediated the relationship between sleep quality and enlarged perivascular spaces severity in the centrum semiovale.

**Conclusion:**

These findings suggest that intravoxel incoherent motion imaging may provide important value for an accurate classification of insomnia. Cerebral microcirculation may have a mediating effect on insomnia and glymphatic system dysfunction.

## Graphical abstract

To explore the relationship among glymphatic system, sleep quality and IVIM parameters in patients with insomnia disorder by using IVIM imaging technology. The SVM classifier based on IVIM information is good for the diagnosis of insomnia. Microperfusion may play a role between insomnia and glymphatic function.

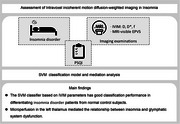

## 1 Introduction

Insomnia disorder (ID) ranks as the predominant sleep disorder and the second leading neuropsychiatric condition following anxiety disorder, impacting 3.9% to 22.1% of people worldwide (Morin et al. 2015, Van Someren 2021). ID is characterized by frequent sleep disturbances or premature awakenings, that occur at least thrice weekly over 3 months, leading to considerable discomfort or daytime dysfunctions (American Psychiatric Association 2013, American Academy of Sleep Medicine 2014). ID may manifest independently or in combination with other medical or mental health disorders, such as Alzheimer's disease (AD), Parkinson's disease, cardiovascular disease, depression, and anxiety disorders (Roth et al. 2006, Sarsour et al. 2010). ID reduces quality of life, imposes a heavy socioeconomic burden, and represents a critical public health issue (Perlis et al. 2022, Huang et al. 2022). ID has been a hot topic of research in recent years. Nevertheless, the neurobiological mechanisms underlying ID are not fully understood.

Neuroimaging, particularly magnetic resonance imaging (MRI), has become a significant part of insomnia research. To data, a range of MRI methods, such as structural, functional, blood flow, and chemical neuroimaging, have been employed to explore abnormal brain changes in individuals with ID (Bae et al. 2023, Chen et al. 2022, Li et al. 2016). A study based on the voxel‐based morphometry (VBM) technique revealed a reduction in GM volume in the thalamus, cerebellum, insula, and parietal cortex after acute sleep deprivation (Dai et al. 2018). A systematic review of diffusion tensor imaging (DTI) findings in patients with primary insomnia revealed that the frontostriatal circuit, frontal thalamus, cortical–cortical network, and limbic system appear to be the main neural networks with microstructural and network changes in PI patients, suggesting that white matter integrity is impaired in patients with insomnia (Sanjari Moghaddam et al. 2021). Previous studies on brain functional connectivity based on resting‐state functional magnetic resonance imaging also revealed that the connectivity of brain network nodes in patients with primary insomnia changed, which specifically manifested as increased connectivity of default mode network (Leerssen et al. 2019), reduced connectivity of salience network (Wei et al. 2020), and reduced connectivity of frontoparietal network (Li et al. 2018). It is suggested that the symptoms of patients with primary insomnia may be related to the intrinsic brain network connectivity. Based on magnetic resonance spectrum (MRS), Park et al. reported that shorter sleep duration was associated with lower GABA levels in the anterior cingulate cortex and medial prefrontal cortex, which was associated with reduced performance in spatial working memory (Park et al. 2020). A sleep‐cerebral blood flow (CBF) correlation study showed that sleep deprivation was associated with further increases of rCBF in bilateral lateral and medial occipital cortex and bilateral insula (Elvsåshagen et al. 2019). Although many neuroimaging studies have described specific neurobiological alterations in ID, unanimous conclusions about the neurophysiology of this disorder have yet to be reached.

Intravoxel incoherent motion (IVIM) diffusion‐weighted imaging was first introduced in 1986, and aims to characterize the minute movements of water molecules and blood microcirculation within capillaries (Le Bihan et al. 1986). According to the IVIM model, diffusion and perfusion can be quantified using multi *b*‐value DWI acquisition, and low *b*‐value provides a higher sensitivity to perfusion. Moreover, IVIM mainly quantifies three parameters: the *D* value reflects real diffusion information, D* value reflects perfusion information, and *f* value reflects the proportion of perfusion effects in total diffusion effect. The clinical application of IVIM imaging has been widely investigated in recent years, reflecting the current trends in neuroimaging research. This trend is particularly evident in the study of central nervous system diseases, such as cerebrovascular diseases and brain tumors (Hu et al. 2015, Detsky et al. 2017, Wong et al. 2017, Bisdas et al. 2013). In our recent prior study, the IVIM technique was used to study the microstructure and microcirculation of AD patients (Xia et al. 2022). Specifically, the AD group showed increased apparent diffusion coefficient (ADC) value in left occipital lobe, increased *D* in left precuneus, right hippocampus, and left occipital lobe and decreased D* in right posterior cingulate gyrus compared with the NC group. The results demonstrated that IVIM imaging is a promising method for the classification of AD and normal control (NC) subjects, and that the IVIM parameters of the precuneus and cerebellum might be effective biomarkers for the diagnosis of AD. Changes in cerebral blood flow and diffusion in ID patients have been described separately in previous studies, but the research results have been inconsistent. Therefore, the IVIM technique was designed to analyze the characteristics of both tissue diffusion and microcirculation in ID patients in this study.

The glymphatic system has recently been identified as a distinctly varied system for waste removal (Jessen et al. 2015, Rasmussen et al. 2018). Importantly, the glymphatic system mainly operates during sleep and remains mostly dormant when awake (Xie et al. 2013). Chronic sleep disturbance is accompanied by altered glymphatic function along enlarged perivascular spaces (Eide et al. 2022). A study utilizing diffusion tensor imaging analysis along the perivascular space (DTI‐ALPS) indicated that patients in their middle and later years suffering from persistent insomnia experience issues with their glymphatic system (Jin et al. 2024). Specifically, patients with chronic insomnia with or without cognitive impairment had a reduced DTI‐ALPS index compared to normal subjects. The function of the glymphatic system function is considered to be related to factors such as arterial pulsation and cerebral perfusion. We speculate that cerebral microcirculation may underlie the link between insomnia and glymphatic system dysfunction. IVIM can generate many measurements simultaneously, including *D*, D*, and *f*. The diffusion parameter *D* reflects the microstructure of the tissue, and a higher *D* indicates less restriction of water diffusion, which represents decreased microstructure integrity (Federau et al. 2012, Le Bihan et al. 1986, Le Bihan et al. 1988). D* and f are parameters about microvascular perfusion, reflecting the state of tissue microcirculation. D* describes the incoherent motion of blood in the microvascular system macroscopically, and *f* describes the fraction of incoherent signal generated from the vascular compartment in each voxel in the total incoherent signal (Federau et al. 2014).

In summary, this study was conducted to evaluate brain microstructure and microcirculation changes in patients with insomnia, explore the classification performance of IVIM parameters in distinguishing insomnia patients from NC subjects, and explore the potential mediating effects of these parameters on insomnia and glymphatic system dysfunction.

## 2 Materials and Methods

### 2.1 Participants

This study was approved by the Institutional Review Board of our hospital. All participants in the examination provided written informed consent. A total of 84 participants were enrolled in this study, including 42 patients with ID (19 males and 23 females, mean age 34.7 years) and 42 NC subjects (16 males and 26 females, mean age 32.5 years). All participants were asked to complete the Pittsburgh Sleep Quality Index (PSQI) and magnetic resonance imaging (MRI). Educational level, height and weight were recorded, and BMI was subsequently calculated.

The inclusion criteria for patients with ID were as follows: (a) aged 18 to 60 years, in junior high school or above; (b) met the diagnostic criteria for insomnia in the Chinese Classification and Diagnostic Criteria of Mental Disorders, Version 3 (CCMD‐3) (Psychosis Branch of Chinese Medical Association, 2001); and (c) had a total PSQI score of greater than or equal to 7 (Zeng et al. 2020). The major exclusion criteria were as follows: (a) secondary insomnia or insomnia caused by lifestyle or environmental changes; (b) psychiatric disorders or other somatic disorders affecting the central nervous system; (c) abnormal liver and kidney function caused by heart, kidney, liver or hematopoietic system diseases; and (d) alcohol abuse, allergies, pregnancy or lactation.

The inclusion criteria for NC participants were as follows: good sleep quality (PSQI ≤5), no history of psychiatric or neurologic diseases, and normal conventional MRI findings.

### 2.2 MRI Acquisition

All examinations were performed on a 3.0 T clinical MRI system (Achieva, Philips, Best, Netherlands) with an 8‐channel head coil. Additionally, to reduce the effects of scanner noise and head motion, all subjects laid in a supine position with ear plugs and foam pads. IVIM images were acquired with 12 *b*‐values (*b* = 0, 10, 25, 50, 75, 100, 150, 200, 400, 800, 1000, and 1500 s/mm^2^) in three orthogonal directions using a single‐shot diffusion‐weighted spin‐echo EPI sequence. The other parameters of IVIM were as follows: repetition time (TR)/echo time (TE) = 3000/114 ms, flip angle = 90°, slice thickness = 5 mm without gap, matrix = 148 × 144, field of view (FOV) = 220 × 220 × 150 (RL/AP/FH) mm^3^, voxel size = 1.49×1.53 mm^2^, reconstruction = 256 × 256, NA = 1, parallel imaging with an acceleration factor of 2, and fat suppression (SPIR). The total acquisition time was 3 min 36 s. Sagittal 3D T1WI was obtained using a fast field echo (FFE) sequence with the following parameters: TR/TE = 8.2/3.8 ms, flip angle = 12°, slice thickness = 1 mm without a gap, matrix size = 256 × 256, FOV = 256 × 256 × 188 mm^3^. The total acquisition time was 4 min 52 s.

The following conventional MRI sequences were included: T1WI FLAIR (TR/TE = 1875/20 ms, inversion time (TI) = 800 ms, slice thickness = 6 mm, slice gap = 1 mm, matrix size = 256 × 136, FOV = 230 × 183 × 139 mm^3^); T2WI TSE (TR/TE = 1885/80 ms, slice thickness = 6 mm, slice gap = 1 mm, matrix size = 256 × 224, FOV = 230 × 190 × 131 mm^3^); T2WI FLAIR (TR/TE = 6000/120 ms, TI = 2200 ms, slice thickness = 6 mm, slice gap = 1 mm, matrix size = 240 × 134, FOV = 230 × 184 × 139 mm^3^); and DWI (TR/TE = 2648/90 ms, flip angle = 90°, slice thickness = 6 mm, slice gap = 1 mm, matrix = 128 × 128, FOV = 230 × 230 × 139 mm^3^, *b* value = 0, 1000 s/mm^2^ along three orthogonal directions).

### 2.3 Postprocessing

IVIM parametric maps were generated using an in‐house MATLAB program (The MathWorks Inc., Natick, MA, USA). Least‐squares fitting of a biexponential model was used for the calculation of IVIM parameters (*D*, D*, and *f*). The equation was as follows:

$$S\left(b\right)/S\;\left(0\right)=f\cdot e^{-b\cdot D*}+\left(1-f\right)\cdot e^{-b\cdot D}$$

**FIGURE 1:**
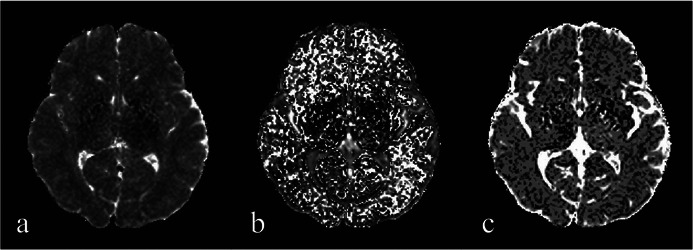
IVIM parameter maps: (a) pure diffusion coefficient (*D*), (b) pseudo diffusion coefficient (D*), (c) perfusion fraction (*f*).

Where *S*(*b*) is the signal intensity at diffusion weighting factor *b*; *S*(0) is the signal intensity at *b* = 0 s/mm^2^; f is the volume fraction of the microcirculation; *D* is the pure diffusion coefficient; D* is the pseudo‐diffusion coefficient. Values with *f* < 0 or *f* > 0.3 were excluded (Xia et al. 2022, Yao et al. 2016). The IVIM parameter map is shown in Figure 1.

### 2.4 Image Analysis

IVIM and 3D T1WI data were preprocessed using FSL (FMRIB, Oxford, UK). This software applies brain extraction and corrections for susceptibility, eddy current and subject movement. IVIM images were registered to 3D T1WI and then to the Montreal Neurological Institute (MNI) template brain, for automatic anatomical segmentation based on the Anatomical Automatic Labeling (AAL90) mask using Advanced Normalization Tools (ANTs). A total of 22 regions were analyzed, including the right and left dorsolateral superior frontal gyrus, hippocampus, anterior cingulate gyrus/ paracingulate cortex, posterior cingulate gyrus, thalamus, precuneus, caudate nucleus, putamen, pallidum, amygdala, and fusiform gyrus (Dai et al. 2014, Riemann et al. 2007, Yan et al. 2018).

### 2.5 Rating of Enlarged Perivascular Spaces

The evaluation was performed according to the criteria of Potter et al. (Potter et al. 2015). The quantity of EPVS in the centrum semiovale and basal ganglia were rated as follows: 0 = no EPVS, 1 ≤10 EPVS, 2 = 10–20 EPVS, 3 = 21–40 EPVS, and 4 ≥40 EPVS. We used an overall score for both hemispheres by assessing and scoring each hemisphere separately and then using the hemisphere with the higher score where the hemispheres were asymmetric. Two neuroradiologists set common criteria, EPVS was scored by a junior radiologist, and then checked by the other senior radiologist.

### 2.6 Statistical Analysis

Statistical analysis was performed in SPSS (Version 22.0, IBM, New York, USA) and R (Version 4.3.2). The caret package in R is used to build SVM models. The SVM model was built using a radial basis function kernel, and the sigma hyperparameter was determined from the estimation based upon the 0.1 and 0.9 quantiles of the samples. The variable importance was calculated by the function of varImp, and the importance ranking was visualized with ggplot2. Normality was tested using the Shapiro–Wilk test. Independent sample t test, Mann–Whitney U tests or chi‐square tests were used for comparisons between groups according to the data type and normal distribution. The data are expressed as means ± standard deviations or *n*(%) according to the type of data.

Considering multiple brain regions, the IVIM parameters between groups were corrected via the false discovery rate (FDR). We subsequently developed a classification model using IVIM parameters to distinguish patients with primary insomnia from NCs. Variables of IVIM parameters with *P* < 0.10 from the group comparison were considered for classification modeling via SVM. The subjects were randomly assigned to the training group (80%) or the validation group (20%). The model was trained on the training set via 10‐fold cross validation to avoid overfitting effect. The optimal model was selected according to the accuracy in this study. And then the established model was tested on the validation dataset. The performance of the model was evaluated via the area under the receiver operating characteristic (ROC) curve (AUC), sensitivity, specificity, and accuracy. The importance of the model features was subsequently ranked.

Alternatively, partial correlation analysis was used to explore the relationships among IVIM parameters, the PSQI score or the MRI‐visual‐EPVS score, with age, gender, educational level and BMI as covariates. Mediation analysis was used to explore the effects of IVIM parameters between the PSQI and MRI‐visual‐EPVS, with age, gender, education and BMI as covariates. Causal mediation analysis was conducted via the ‘mediation’ package in R 4.3.2. The bootstrap method with 500 simulations was used to estimate indirect and direct effects.

## 3 Results

### 3.1 Demographic and Clinical Data Results

The demographic characteristics and clinical data are presented in Table 1. There were no significant differences in gender (*p* = 0.507), age (*p* = 0.212), or BMI (*p* = 0.929) between the two groups. The ID group had a lower level of education than the NC group did (*p* = 0.034). The PSQI score was significantly greater in the insomnia group than in the control group (*p* < 0.001).

**TABLE 1:** Demographic characteristics of the insomnia and normal groups.

	ID(*N* = 42)	NC(*N* = 42)	*p* value
Gender			0.507
Male	19(45.2%)	16(38.1%)	
Female	23(54.8%)	26(61.9%)	
Age (year)	34.69±9.59	32.45±10.51	0.212
Education (year)	13.71±3.34	15.17±3.29	0.034
BMI (kg/m^2^)	21.73±2.88	21.79±2.95	0.929
Total scores	12.36±2.84	3.45±1.33	<0.001
BG‐EPVS			<0.001
0	0(0%)	1(2.4%)	
1～10	2(4.8%)	9(21.4%)	
11～20	26(61.9%)	29(69.0%)	
21～40	13(31.0%)	3(7.1%)	
>40	1(2.4%)	0(0%)	
CSO‐EPVS			0.036
0	0(0%)	6(14.3%)	
1～10	20(47.6%)	21(50.0%)	
11～20	10(23.8%)	8(19.0%)	
21～40	9(21.4%)	5(11.9%)	
>40	3(7.1%)	2(4.8%)	

### 3.2 Imaging Characteristics

**FIGURE 2:**
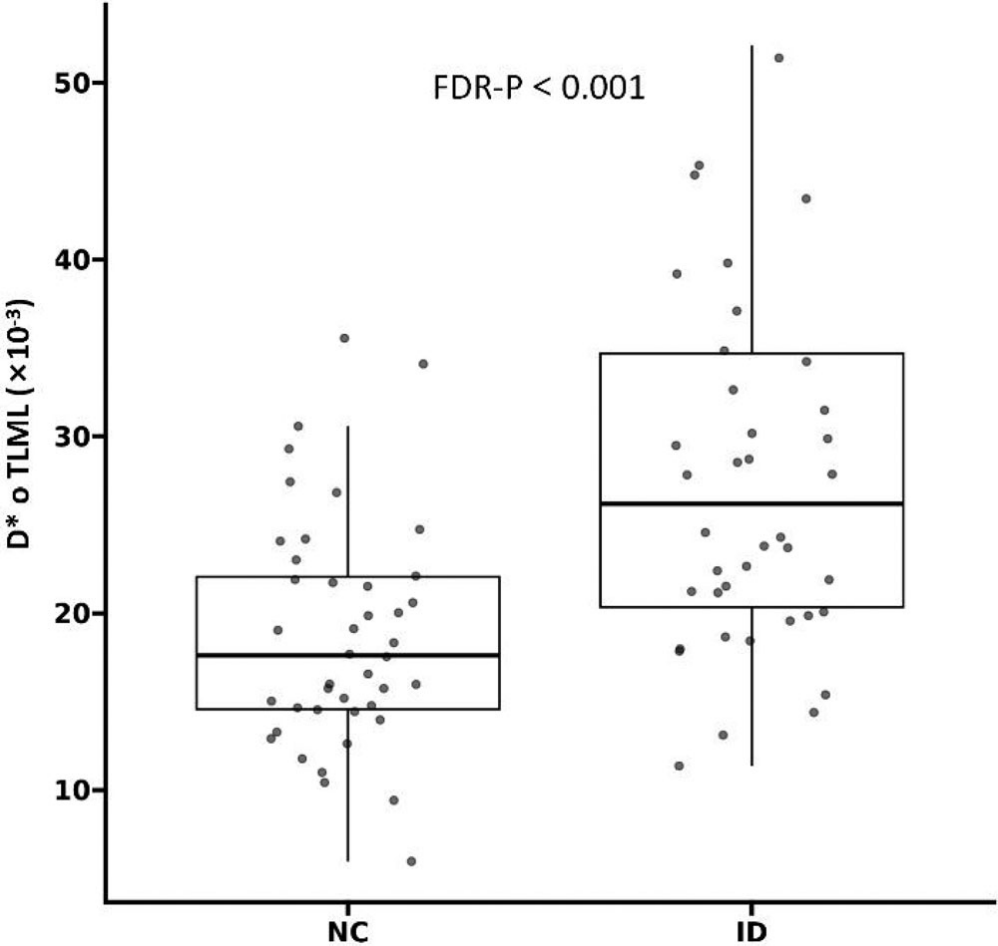
Boxplots of D* of TLML for NC and ID groups.

The differences in the IVIM parameters between the two groups were mainly reflected in the perfusion parameters (D* and *f*), as shown in Table 2. Overall, microcirculation increased in the left brain region and decreased in the right brain region. The D* values of HPCL, PTML and TLML, and the *f* values of HPCL, FSFL, PTML and PLDL in the ID group were greater than those in the healthy group (FDR‐*p* < 0.05). The D* values of DSFCR, CADR, PTMR and PLDR, and the f values of DSFCL, DSFCR, and AMGR were lower in the ID group than in the healthy group (FDR‐*p* < 0.05). In addition, MRI‐visible EPVS in the basal ganglia (*p* < 0.001) and centrum semiovale (*p* = 0.036) were more severe in the ID group than in the NC group. Figure 2 shows the boxplot distribution of D* of TLML in the two groups.

**TABLE 2:** IVIM features of insomnia and normal groups.

	ID(*N* = 42)	NC(*N* = 42)	*Z* or *t* value	FDR corrected
D*(10^−3^)				
DSFCR	20.20±4.66	23.34±5.19	*t* = 2.916	0.027
HPCL	41.98±15.33	31.81±19.09	*Z* = ‐4.079	0.001
CADR	34.38±14.07	41.82±13.93	*Z* = ‐2.782	0.027
PTML	39.83±14.37	29.33±12.52	*Z* = ‐3.623	0.005
PTMR	53.35±25.43	66.06±17.59	*Z* = ‐3.238	0.011
PLDR	34.46±23.44	47.88±20.93	*Z* = ‐3.417	0.008
TLML	28.65±11.19	18.79±6.54	*Z* = ‐4.357	<0.001
f				
DSFCL	0.14±0.02	0.15±0.02	*Z* = ‐2.809	0.027
DSFCR	0.15±0.02	0.17±0.01	*Z* = ‐3.106	0.028
HPCL	0.20±0.02	0.18±0.02	*t* = ‐1.542	0.019
AMGR	0.18±0.04	0.20±0.05	*t* = 2.623	0.040
FSFL	0.17±0.02	0.16±0.02	*t* = ‐3.369	0.011
PTML	0.12±0.01	0.10±0.02	*Z* = ‐4.804	<0.001
PLDL	0.11±0.05	0.07±0.07	*t* = ‐3.330	0.004

### 3.3 SVM Classification Model

**FIGURE 3:**
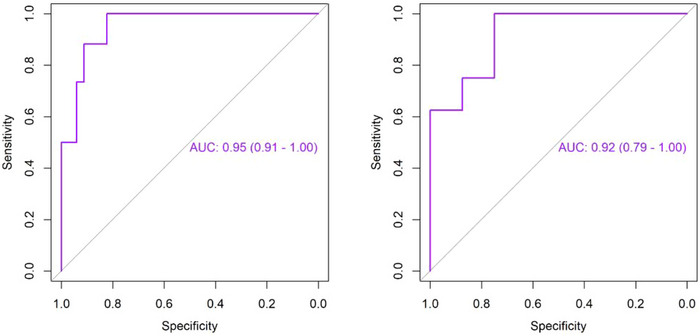
ROC curves of the SVM classification model based on IVIM parameters.

**FIGURE 4:**
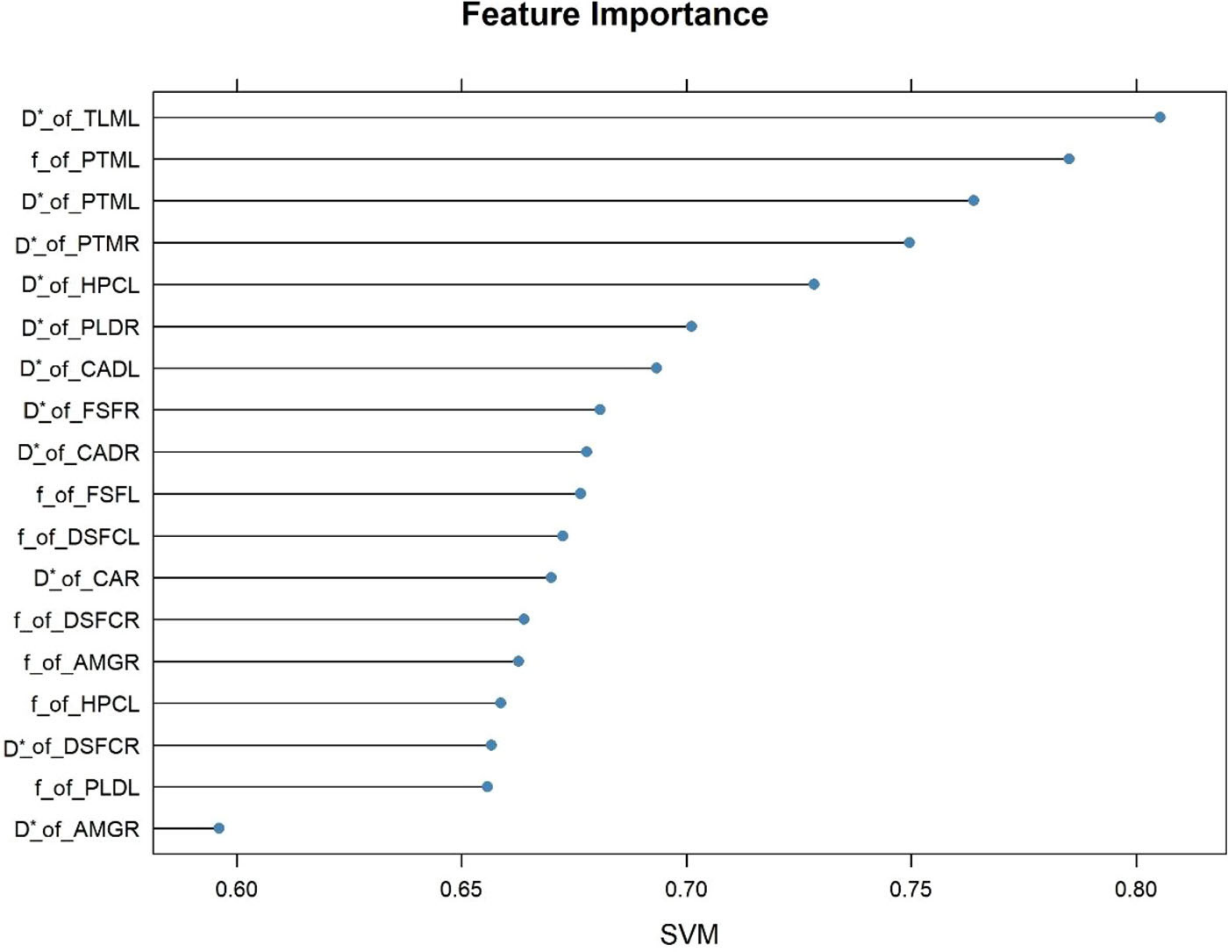
Model feature importance ranking.

As shown in Table 3 and Figure 3, the SVM model based on IVIM parameters has a good diagnostic efficiency for insomnia in general. In the testing set, an accuracy of 81.25% was obtained via the IVIM‐based SVM classifier to distinguish ID from NC, with a sensitivity of 62.50%, a specificity of 100.00%, and an AUC of 0.92. Figure 4 shows the feature importance ranking of the SVM model, and it can be seen that the D* of TLML has a large contribution to classification.

**TABLE 3:** Performance metrics of SVM classification for ID using IVIM parameters.

	AUC(95%CI)	Accuracy	Sensitivity	Specificity
Train	0.95(0.91,1,00)	0.87	0.82	0.91
Test	0.92(0.79,1.00)	0.81	0.63	1.00

### 3.4 Partial Correlation Analysis

**FIGURE 5:**
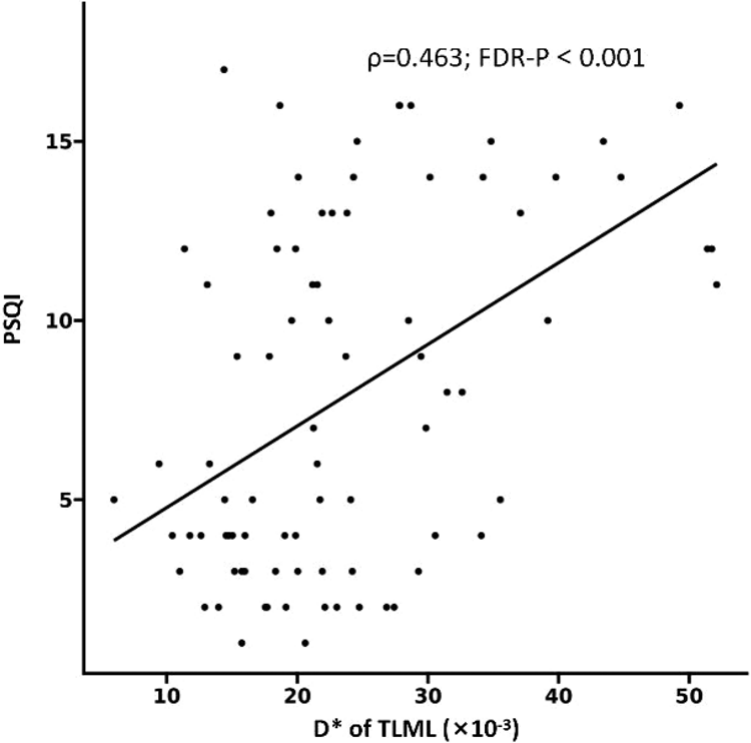
Scatter plot of D* of TLML versus PSQI.

As shown in Table 4, 11 IVIM parameters were significantly associated with the PSQI score after adjusting for age, sex, years of education, and BMI. Specifically, the D* of HPCL (*ρ* = 0.303; fdr‐*p* = 0.016), PTML (*ρ* = 0.321; fdr‐*p* = 0.013), and TLML (*ρ* = 0.463; fdr‐*p* < 0.001), and the f of HPCL (*ρ* = 0.247; fdr‐*p* = 0.045), FSFL (*ρ* = 0.291; fdr‐*p* = 0.020), PTML (*ρ* = 0.466; fdr‐*p* < 0.001), and PLDL (*ρ* = 0.402; fdr‐*p* = 0.016) were positively correlated with the PSQI score. The D* of DSFCR (*ρ* = −0.366; fdr‐*p* = 0.005), CAR (ρ = −0.316; fdr‐*p* = 0.013), CADR (*ρ* = −0.261; fdr‐*p* = 0.035), and the *f* of DSFCR (*ρ* = −0.276; fdr‐*p* = 0.027) were negatively correlated with the PSQI score. Figure 5 shows a scatter plot of D* of TLML versus PSQI.

**TABLE 4:** Partial correlation analysis between IVIM parameters, PSQI scores, and EPVS (with age, gender, BMI, and years of education as covariates).

		Partial correlation statistics	
		ρ FDR corrected	
**IVIM parameters**	**PSQI scores**		
D* of DSFCR		−0.366	0.005
D* of CAR		−0.316	0.013
D* of HPCL		0.303	0.016
D* of CADR		−0.261	0.035
D* of PTML		0.321	0.013
D* of TLML		0.463	<0.001
*f* of DSFCR		−0.276	0.027
*f* of HPCL		0.247	0.045
*f* of FSFL		0.291	0.020
*f* of PTML		0.466	<0.001
*f* of PLDL		0.402	0.016
**IVIM parameters**	**EPVS**		
D* of TLML	CSO‐EPVS	0.338	0.0039
**EPVS**	**PSQI scores**		
BG.EPVS	PSQI	0.423	<0.001
CSO.EPVS	PSQI	0.234	0.036

With respect to the MRI‐visible EPVS, the D* of the TLML was positively correlated with the severity of the CSO‐EPVS (*ρ* = 0.338; fdr‐*p* = 0.039). In addition, BG‐EPVS (*ρ* = 0.423; fdr‐*p* < 0.001) and CSO‐EPVS (*ρ* = 0.234; fdr‐*p* = 0.036) were positively correlated with PSQI score.

### 3.5 Mediation Analysis

In the mediation analysis, the D* of TLML (indirect effect *β* = 0.030, *p* = 0.016) had a significant mediating effect on the relationship between the PSQI score and CSO‐EPVS, as shown in Table 5. However, we did not observe a mediating effect of the D* of the TLML on the relationship between the PSQI score and BG‐EPVS. In addition, we did not find a mediating effect of the D* of HPCL between the PSQI and the BG‐EPVS or CSO‐EPVS.

**TABLE 5:** Analysis of mediation (with age, gender, BMI, and years of education as covariates).

mediating variable	Dependent variable	Total effect		Indirect effect		Direct effect	
		Coefficient(95%CI)	*p* value	Coefficient(95%CI)	*p* value	Coefficient(95%CI)	*p* value
D* of TLML	CSO‐EPVS	0.05(0.00,0.11)	0.044	0.03(0.01,0.06)	0.016	0.02(‐0.02,0.09)	0.396

## 4 Discussion

In the present study, the IVIM technique was used to investigate the microstructure and microcirculation of ID patients. Overall, the perfusion parameters of IVIM (D* and *f*) in multiple brain regions were altered in ID patients. The SVM classifier, which is based on IVIM parameters, has good classification performance in differentiating ID patients from NCs. Microperfusion in the left thalamus mediated the relationship between insomnia and glymphatic system dysfunction.

CBF is an index that reflects cerebral activity and the metabolic rate. The association between sleep and CBF has been previously demonstrated. According to the synaptic homeostasis theory, CBF decreases during sleep and increases during wakefulness (Elvsåshagen et al. 2019, Tononi and Cirelli 2014). The findings of the present study revealed changes (increases and decreases) in microperfusion but not in tissue diffusion in multiple brain regions associated with insomnia. Similar to previous studies, this study also provided some clues to the cerebral blood flow disorder in patients with sleep disorders. Unlike previous studies, the D* and *f* parameters of IVIM provide information related to the diffusion of water molecules in the blood and blood fraction (Federau et al. 2014). Microcapillary perfusion in different brain regions increased or decreased inconsistently. Overall, microcirculation increased in the left brain region and decreased in the right brain region. This may be an interesting phenomenon and probably corresponds to the lateralization of brain structure and function. Previous studies have also demonstrated that there are differences in the structure and function among multiple brain regions in the left right handedness population (Ocklenburg and Guo 2024, Qin et al. 2024). Therefore, it is necessary to consider the characteristics of brain laterality when studying the changes in brain regions in patients with insomnia. An imbalance of electroencephalographic (EEG) activity between brain hemispheres has been suggested to occur in individuals with ID (St‐Jean et al. 2012). A meta‐analysis revealed a significant increase in brain activity in the left superior temporal gyrus and the right superior longitudinal fasciculus in patients with ID, whereas brain activity was reduced in multiple regions of the right hemisphere (Duan et al. 2023). Insomnia is typically accompanied by a decrease in CBF (Huang et al. 2022, Yan et al. 2023). However, regional blood flow may increase in some regions in the early stages of some diseases, which is associated with compensatory changes in neural activity. Importantly, the participants in this study were young (Dai et al. 2009). In addition, we did not find differences in the D between the two groups, which was inconsistent with most previous studies of diffusion imaging in insomnia (Grau‐Rivera et al. 2020, Cai et al. 2019). However, several studies have shown that no significant difference in ADC exists between sleep‐deprived people and rested people (Demiral et al. 2019).

At present, the diagnosis of insomnia primarily relies primarily on history collection, which is based on the subjective judgment of patients and doctors (Sutton 2021). However, objective measures such as polysomnography (PSG) and actigraphy are not routinely recommended because of their intrinsic drawbacks, such as limitations in detecting specific sleep disturbances for actigraphy and tolerance and convenience for PSG (Sutton 2021). Other objective techniques, such as neuroimaging, particularly MRI, will undoubtedly play a role in insomnia research. In addition, the combination of MRI techniques with machine learning algorithms could provide more useful information for disease classification. In a recent study, He et al. (He et al. 2022) directly diagnosed ID via objective criteria of fMRI‐based whole‐brain functional connectivity with the SVM method, and the SVM classifier achieved good classification performance (AUC = 0.893). Wen et al. (Wen et al. 2023) also diagnosed the ID of the hemodialysis population via fMRI‐based fractional amplitude of low frequency fluctuation (fALFF) with the SVM method, and the SVM classifier achieved good classification performance (AUC = 0.8202). Similarly, the results of this study also demonstrated good classification performance for ID patients when the IVIM technique was combined with the SVM classification model (AUC = 0.92). Different modalities of MRI may contain complementary information for the diagnosis of ID. Therefore, future studies should be designed with multimodal imaging including IVIM to further improve the diagnostic accuracy for ID.

The thalamus is related to the neurobiological mechanism of insomnia, and it plays a crucial role in regulating the sleep‐wake cycle, hyperarousal activity, and emotional state, and restoring autonomic nervous and endocrine functions (Huang et al. 2022, Li et al. 2019). Various types of neuroimaging studies have also shown that thalamic abnormalities exist in individuals with sleep disorders. Shokri et al. performed PET/CT examinations on 20 healthy subjects after a night of sleep deprivation and reported significant accumulation of β‐amyloid in the right hippocampus and thalamus (Shokri‐Kojori et al. 2018). Patients with ID have increased connectivity between the left thalamus and the right precentral gyrus, the right thalamus and the left middle frontal gyrus, and the right superior parietal lobule and the right superior frontal gyrus (Huang et al. 2022). A recent study reported an increase in gray matter CBF in the thalamus, hippocampus, and amygdala after 1 day of nocturnal wakefulness and sleep deprivation and a decrease in gray matter CBF after adequate sleep (Tononi and Cirelli 2014, Wu et al. 2006).

In the present study, our findings suggested that microperfusion in the left thalamus mediated the relationship between insomnia and glymphatic system dysfunction. Recent studies have demonstrated that neural activity can alter CSF flow via neurovascular coupling (Fultz et al. 2019, Williams et al. 2023). In addition, Susanne et al. suggested that low frequency arteriolar oscillations drive drainage of solutes which is hypothesized to be one of the drivers of perivascular glymphatic convective transport of solutes (Van Veluw et al. 2020). A recent study suggested that sleep deprivation may impair cognitive function through hemodynamic changes associated with neurovascular coupling (Csipo et al. 2021). The neurovascular coupling promotes not only the supply of metabolites but also the removal of metabolic waste products (Holstein‐Rønsbo et al. 2023). Therefore, it is reasonable to speculate that thalamus may mediate insomnia and glymphatic system dysfunction via neurovascular coupling.

There were several limitations in our study. First, the sample size for ID patients was not large, and future studies with larger sample sizes are needed for analysis. Second, this is a cross‐sectional study, and the inference value is limited. The relationships among microperfusion, insomnia and glymphatic system dysfunction must be further confirmed by more studies. Third, the impact of insomnia duration and severity on the research results was not considered in this study. Fourth, although IVIM can detect microcirculation perfusion without contrast agent, the repeatability of perfusion related parameters is insufficient, which needs to be further optimized by improving the scanning protocol and algorithm.

In conclusion, IVIM imaging is a promising method for objectively evaluating the brain tissue structure and microcirculation in patients with ID, and it is a suitable and useful technique for differentiating ID patients from NCs. The SVM classification model, which is based on IVIM parameters, has good diagnostic performance. Regional cerebral microperfusion has a mediating effect on insomnia and glymphatic system dysfunction, which may provide a theoretical basis for the prevention and treatment of insomnia.

## Nomenclature

IVIM: Intravoxel incoherent motion
*D*
: Pure diffusion coefficient
D*
: Pseudo diffusion coefficient
*f*
: Perfusion fraction
SVM: Support vector machine
AUC: Area under the receiver operating characteristic curve
DSFCL: Left Dorsolateral superior frontal cortex
DSFCR: Right dorsolateral superior frontal cortex
CAL: Left anterior cingulate cortex and paracingulate cortex
CAR: Right anterior cingulate cortex and paracingulate cortex
PCCL: Left posterior cingutate cortex
PCCR: Right posterior cingutate cortex
HPCL: Left hippocampus
HPCR: Right hippocampus
AMGL: Left amygdala
AMGR: Right amygdala
FSFL: Left fusiform
FSFR: Right fusiform
PCSL: Left precuneus
PCSR: Right precuneus
CADL: Left caudate
CADR: Right caudate
PTML: Left putamen
PTMR: Right putamen
PLDL: Left pallidum
PLDR: Right pallidum
TLML: Left thalamus
TLMR: Right thalamus

## Author Contributions

**Wenjie Tang**: methodology, formal analysis, writing–original draft. **Asta Debora**: resources, validation. **Shouliang Miao**: data curation. **Boli Lin**: conceptualization, investigation. **Qun Huang**: validation, software. **Zhengzhong Yuan**: Funding acquisition, supervision. **Yunjun Yang**: Supervision, project administration. **Nengzhi Xia**: Supervision, writing–review and editing.

## Conflicts of Interest

The authors declare no conflicts of interest.

## Peer Review

The peer review history for this article is available at https://publons.com/publon/10.1002/brb3.70785.

## Data Availability

Data sharing is not applicable to this article as no new data were created or analyzed in this study.
